# Systemic Pharmacotherapeutic Treatment of the ACTA1-MCM/FLExDUX4 Preclinical Mouse Model of FSHD

**DOI:** 10.3390/ijms25136994

**Published:** 2024-06-26

**Authors:** Ngoc Lu-Nguyen, Stuart Snowden, Linda Popplewell, Alberto Malerba

**Affiliations:** 1 Department of Biological Sciences, School of Life Sciences and the Environment, Royal Holloway University of London, Egham, Surrey TW20 0EX, UK; ngoc.lu-nguyen@rhul.ac.uk (N.L.-N.); stuart.snowden@rhul.ac.uk (S.S.); 2 National Horizons Centre, Teesside University, Darlington DL1 1HG, UK

**Keywords:** FSHD, DUX4 inhibition, berberine, ACTA1-MCM/FLExDUX4 mice

## Abstract

Aberrant expression of the double homeobox 4 (*DUX4*) gene in skeletal muscle predominantly drives the pathogenesis of facioscapulohumeral muscular dystrophy (FSHD). We recently demonstrated that berberine, an herbal extract known for its ability to stabilize guanine–quadruplex structures, effectively downregulates *DUX4* expression in FSHD patient-derived myoblasts and in mice overexpressing exogenous *DUX4* after viral vector-based treatment. Here, we sought to confirm berberine’s inhibitory efficacy on *DUX4* in the widely used FSHD-like transgenic mouse model, ACTA1-MCM/FLExDUX4, where *DUX4* is induced at pathogenic levels using tamoxifen. Animals repeatedly treated with berberine via intraperitoneal injections for 4 weeks exhibited significant reductions in both mRNA and protein levels of *DUX4*, and in mRNA expression of murine DUX4-related genes. This inhibition translated into improved forelimb muscle strength and positive alterations in important FSHD-relevant cellular pathways, although its impact on muscle mass and histopathology was less pronounced. Collectively, our data confirm the efficacy of berberine in downregulating *DUX4* expression in the most relevant FSHD mouse model. However, further optimization of dosing regimens and new studies to enhance the bioavailability of berberine in skeletal muscle are warranted to fully leverage its therapeutic potential for FSHD treatment.

## 1. Introduction

Despite being the third most common type of muscular dystrophy, there are no disease-modifying treatments available for facioscapulohumeral muscular dystrophy (FSHD). The molecular hallmark of the disease is the loss of epigenetic features from the D4Z4 macrosatellite repeats located in the subtelomeric region of chromosome 4q35 [1], particularly in the presence of a permissive 4qA haplotype. Each D4Z4 unit contains an incomplete copy of the double homeobox 4 (*DUX4*) gene, encoding the transcription activator DUX4, predominantly expressed during germline and early embryonic stages but silenced in adult somatic tissues, including muscle [2]. The deletion of a large region of the D4Z4 repeat array to 1–10 units [3], prevalent in the majority of FSHD patients (classified as FSHD1), induces chromatin relaxation and extensive hypomethylation, leading to transcription of *DUX4* from the most distal D4Z4 repeat [3]. FSHD2, the second form of the disease, also involves *DUX4* mis-expression, primarily due to mutations in epigenetic modulators such as the structural maintenance chromosome flexible hinge domain containing 1, *SMCHD1* [4], or the DNA methyltransferase 3B, *DNMT3B* [5]. In both FSHD1 and FSHD2, aberrant *DUX4* expression in mature skeletal muscles dysregulates numerous cellular pathways, triggering inflammatory responses, reducing antioxidant defenses, and preventing muscle regeneration, contributing to muscle fibrosis and fatty infiltration [6,7,8,9]. Notably, the increased susceptibility to D4Z4 hypomethylation, associated with a shorter D4Z4 array in FSHD1 and/or *SMCHD1* mutations in FSHD2, significantly impacts the disease severity, underscoring the intricate interplay between genetic and epigenetic imbalances in FSHD development [10,11]. Despite this, the complex mechanisms underlying the disease remain elusive. 

The D4Z4 repeat array, with approximately 73% GC content, is believed to harbor biologically significant epigenetic features [12], suggesting that high levels of CpG methylation, along with repressive histone modifications, may contribute to chromatin repression in somatic cells [13]. Additionally, secondary nucleic structures known as G-quadruplexes (GQs) have been identified within the D4Z4 array [14,15]. These GQs are formed within G-rich DNA and RNA sequences and consist of G-quartets held together by Hoogsten hydrogen bonding in a planar orientation [16]. It is now well established that GQ structures, usually located in the promoters, enhancers, telomeres, transcripts, and non-coding RNAs [17,18], are important regulatory elements and that they hold important roles in various cellular functions linked to DNA regulation and RNA post-transcriptional mechanisms [19].

We have identified the presence of novel GQ motifs within the enhancer, promoter, and transcript of *DUX4* and have demonstrated that berberine, an herbal extract, exhibits a high binding affinity to these DNA and RNA GQ structures [20]. Interaction of berberine with the GQs is predicted to stabilize the G-quartets, thereby suppressing expression of *DUX4*. Indeed, we have proven that berberine treatment in FSHD patient-derived muscle cells and in mice, injected intramuscularly with adeno-associated viral vectors expressing exogenous *DUX4*, decreased expression of *DUX4* and its downstream targets, and improved muscle histopathology and muscle strength of tibialis anterior (TA) muscle, compared to untreated controls [20]. Given the increasing popularity of the double-transgenic ACTA1-MCM/FLExDUX4 mouse model for studying FSHD and its important similarities to patient phenotypes (i.e., disease-relevant low and stochastic *DUX4* expression, progressive muscle wasting, reduction in muscle strength, and elevation in muscle fibrosis) [21,22], we sought to confirm berberine’s efficacy in inhibiting *DUX4* in these transgenic mice. Our study involved a four-week systemic treatment of berberine, administered via repeated intraperitoneal injections. We report here our findings from analyses of body-wide muscle mass and muscle functionality, as well as molecular and histopathological assessments in TA muscle.

## 2. Results

### 2.1. Berberine Treatment Has Minimal Effect on Muscle Mass and Other Muscle Functions but Improves Forelimb Muscle Strength

**Figure 1:**
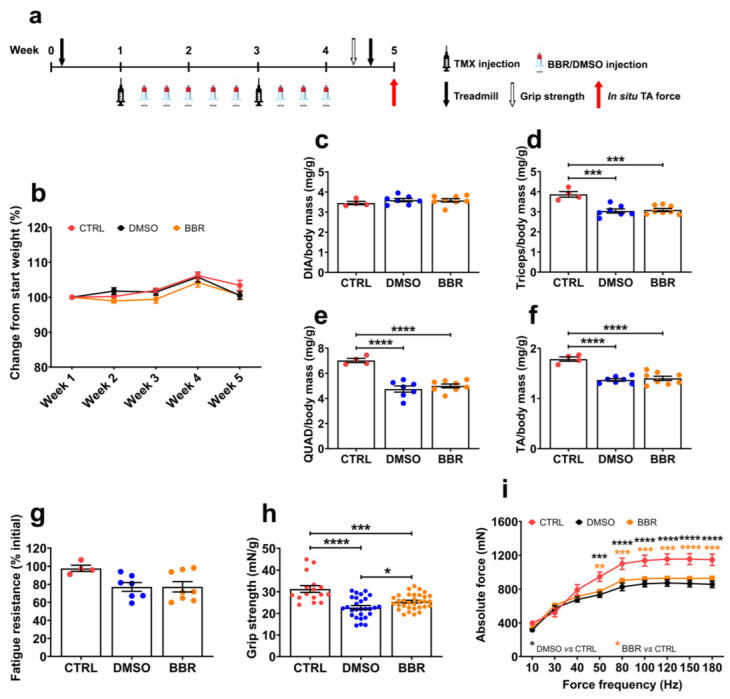
Effect of berberine treatment on muscle mass and muscle functionality. (**a**) Diagram of the experimental design, including timepoints of injections of tamoxifen (TMX), treatment of berberine (BBR) or DMSO, and functional tests. ACTA1-MCM/FLExDUX4 mice received 2.5 mg/kg/injection of TMX via intraperitoneal (IP) delivery on weeks 1 and 3 to induce *DUX4* expression. The mice were further IP injected with 8 × 10 mg/kg/injection of BBR (*n* = 8) or DMSO (*n* = 7), over a 4-week period. ACTA1-MCM mice receiving volume-matched saline via IP were considered as a healthy control (CTRL, *n* = 4). (**b**) Weekly bodyweight is shown as the percentage of the initial weight recorded on the day of the first TMX injection. Post-mortem mass of (**c**) diaphragm (DIA), (**d**) triceps, (**e**) quadriceps (QUAD), and (**f**) tibialis anterior (TA) muscles normalized to the corresponding final bodyweight are shown. (**g**) Treadmill exhaustion tests were conducted 1 week before the first TMX injection and repeated 3 days before tissue harvest. Fatigue resistance level is shown as the percentage of the running time in the final test as of the initial running time. (**h**) The forelimb grip strength is shown as the grip force corrected to the bodyweight recorded at the assessment. (**i**) In situ absolute force of TA muscle was measured with increasing force frequency. All data are shown as mean ± S.E.M. Statistical analysis was by one-way (**b**–**g**) or two-way (**a**,**h**) ANOVA followed by Tukey’s multiple comparisons test, * *p* < 0.05, ** *p* < 0.01, *** *p* < 0.001, **** *p* < 0.0001.

In order to asssess the effect of berberine in the ACTA1-MCM/FLExDUX4 mice, two groups of ACTA1-MCM/FLExDUX4 and one group of ACTA1-MCM mice were involved. The ACTA1-MCM/FLExDUX4 double transgenic model carries an inverted human DUX4 sequence inherited from FLExDUX4 mice and tamoxifen (TMX)-inducible Cre recombinase inherited from ACTA1-MCM mice [21]. DUX4 expression was induced by administering TMX at a dose of 2.5 mg/kg/injection every two weeks via intraperitoneal (IP) delivery. Following the first TMX administration, ACTA1-MCM/FLExDUX4 mice were further injected IP with either 10 mg/kg/injection of berberine (BBR, *n* = 8) or volume-matched 5% DMSO (*n* = 7). ACTA1-MCM healthy mice (*n* = 4) receiving volume-matched saline were considered as the healthy control (CTRL). Dosing with berberine, DMSO, or saline was repeated for a total of eight injections over 4 weeks. Details of experimental design are shown in Figure 1a. Bodyweight recorded during this study and normalized to the initial weight showed no difference between animal groups (Figure 1b). However, at the individual muscle level, the mass of the triceps, quadriceps, and tibialis anterior (TA) muscles in both BBR and DMSO groups was significantly reduced, compared to the CTRL values, whereas the diaphragm mass was unaffected (Figure 1c–f). We did not observe any modification in the muscle mass of BBR-treated mice relative to DMSO values. 

To evaluate the impact of DUX4 toxicity and the effect of BBR treatment on whole-body muscle function, treadmill exhaustion tests were conducted. Mice were allowed to run on a treadmill until they were unable to move from the stopper for 10 s. The fatigue resistance level shown in Figure 1g was calculated as the total running time performed at the end of the treatment as a percentage of the time recorded in the initial test. Following two doses of TMX, both BBR- and DMSO-treated mice could resist fatigue by 77% of the initial level, versus 98% in CTRL mice, indicating the negative impact of DUX4 expression in the two ACTA1-MCM/FLExDUX4 groups, although no significant level was observed (*p* = 0.0523). Additional assessment on the strength of the forelimb muscle using a grip strength test demonstrated more obvious impact of DUX4 pathology. While forelimb force in DMSO mice dropped by 29% from 31.3 mN/g of the CTRL value to 22.7 mN/g (*p* < 0.0001), the strength in BBR-treated mice remained at 25.5 mN/g, significantly higher, by 12%, than the DMSO value (*p* = 0.0127) (Figure 1h). Further in situ TA force measurement displayed pronounced muscle weakness in both ACTA1-MCM/FLExDUX4 groups compared to the CTRL group. Despite improving forelimb muscle strength, BBR treatment was insufficient in preventing functionality loss in the examined hindlimb muscle (Figure 1i).

### 2.2. BBR Treatment Efficiently Inhibits mRNA and Protein Expression of DUX4 and Its Downstream Targets

**Figure 2:**
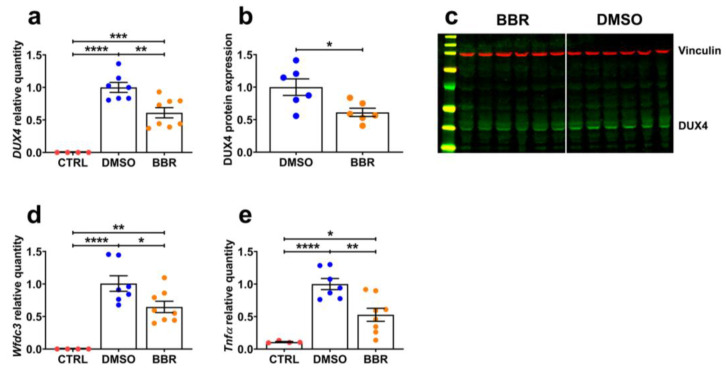
Effect of BBR on expression of *DUX4* and murine DUX4-related genes in TA muscle. (**a**) Following TMX-induced *DUX4* expression and 4-week treatment with BBR, mRNA level of *DUX4* was quantified using qRT-PCR as relative to corresponding *Gapdh* level and expressed as fold-change in the DMSO values obtained in the same way. (**b**) DUX4 protein expression was assessed using Western blotting and expressed as fold-change relative to the DMSO level. (**c**) Representative immunoblotting showing inducible *DUX4* expression (green), with each lane displaying a biological replicate; vinculin (red) was used as a loading control. mRNA expression of (**d**) murine DUX4-target *Wfdc3* and (**e**) pro-inflammatory marker *Tnfα* was quantified using qRT-PCR in the same way as for *DUX4* mRNA. Data are shown as mean ± S.E.M. Statistical analysis was conducted via one-way ANOVA (**a**,**d**,**e**) followed by Tukey’s multiple comparisons test or by Student’s *t* test (**b**), * *p* < 0.05, ** *p* < 0.01, *** *p* < 0.001, **** *p* < 0.0001.

We decided to focus our investigation on the effect of BBR on the TA muscle, despite the observed lack of improvement in functionality, as this muscle type has been studied extensively in preclinical FSHD research [23,24,25,26], and *DUX4* transgene recombination in the TA matches what is seen in most muscles/organs characterized by the Jones Lab [22]. As anticipated, *DUX4* expression in the DMSO group was highly induced, compared to the CTRL value (*p* < 0.0001), and BBR treatment resulted in a 40% reduction in mRNA levels (*p* = 0.0033) (Figure 2a). This reduction corresponded to a comparable decrease of 39% (*p* = 0.0213) in protein expression (Figure 2b,c). Consequently, the mRNA expression of a murine DUX4 downstream target, *Wfdc3* [22], was downregulated by 35% (*p* = 0.0361), compared to the level seen in the DMSO-treated control (Figure 2d). In agreement with previous findings suggesting that DUX4 triggers inflammation [22,27], we observed a significant increase relative to CTRL mice (*p* < 0.0001) in the level of the pro-inflammatory cytokine, *Tnfα*, in the DMSO group, which was reduced by 47% (*p* = 0.0032) with BBR treatment (Figure 2e). These results confirm the efficacy of systemic BBR delivery in down-regulating *DUX4* expression at both mRNA and protein levels, as well as its impact on expression of murine DUX4-related genes.

### 2.3. Insufficient Improvement in TA Muscle Histopathology following BBR Administration

**Figure 3:**
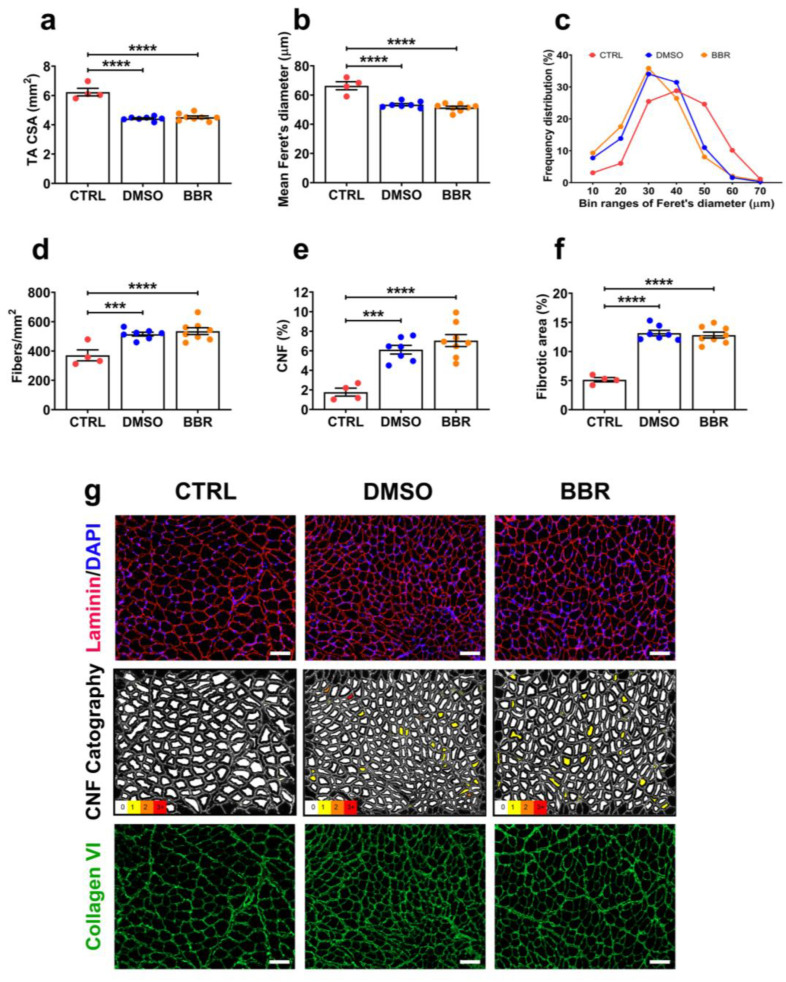
Effect of BBR on DUX4-mediated histopathology in TA muscle. (**a**) Cross-sectional area (CSA) of whole TA muscle was approximated mathematically by dividing the muscle mass by the optimum fiber length and the density of mammalian muscle. (**b**–**g**) Frozen muscle sections were stained for laminin, collagen VI, and DAPI. (**b**) Mean of minimal Feret’s diameter of fibers, (**c**) frequency distribution of the fiber diameter, and (**d**) total fiber numbers per mm^2^ of the muscle CSA are shown, with values in b and d automatically scored using Fiji/MuscleJ software (https://fiji.sc/). (**e**) The total number of centrally nucleated myofibers (CNFs) counted automatically is expressed as a percentage of the total myofiber number within the same transverse muscle section. (**f**) Fibrotic area was semi-automatically evaluated and expressed as percentage of the area positive for collagen VI of the muscle area. Data are shown as means ± S.E.M. Statistical comparison was with one-way ANOVA followed by Tukey’s multiple comparisons test, *** *p* < 0.001, **** *p* < 0.0001. (**g**) Representative images co-immunostained with laminin (red) and DAPI (blue), or with collagen VI (green), are shown at a magnification of ×100, scale bar = 100 µm, as well as cartographic images for CNFs generated with Fiji/MuscleJ, with fibers having 0, 1, 2, and 3+ color-coded as white, yellow, orange, and red, respectively.

The impact of inducible DUX4 expression on TA muscle was further assessed at the histological level. As anticipated, we observed significant reductions in the cross-sectional area (CSA) and correspondingly in the myofiber diameter of TA muscle in both DMSO and BBR groups, relative to the CTRL values (*p* < 0.0001), with no amelioration detected following BBR treatment (Figure 3a,b). Further examination revealed a shift in the myofiber population, characterized by an increased number of small fibers and decreased numbers of large fibers in the two ACTA1-MCM/FLExDUX4 groups, compared to the CTRL properties (Figure 3c). Associated with these changes, we measured an increase in the number of myofibers per mm^2^ in DMSO and BBR muscles (Figure 3d), suggesting that reductions in CSA (Figure 3a) and muscle mass (Figure 1e) may be attributed to DUX4-induced muscle deterioration and involvement of murine innate muscle regeneration, rather than a consequence of muscle atrophy. Indeed, we detected a three-fold increase in the number of centrally nucleated fibers (CNFs), a hallmark of muscle regeneration [28], in both DMSO (*p* = 0.004) and BBR (*p* < 0.0001) muscles, relative to CTRL value (Figure 3e). In addition to muscle wasting, excessive muscle fibrosis negatively affects muscle function and is another important hallmark in FSHD [29]. We therefore examined the level of fibrosis in the TA muscle based on immunostaining for a commonly used marker of fibrosis, collagen VI [30], and identified a significant increase in the fibrotic area, from 5.1% in CTRL muscle to 13.2% in DMSO (*p* < 0.0001) or 12.8% in BBR tissue (*p* < 0.0001) (Figure 3f). BBR treatment did not provide an amelioration in any of the pathological features examined. Representative images from TA histological assessments are shown in Figure 3g. 

### 2.4. BBR Treatment Positively Modifies Important DUX4-Related Cellular Pathways 

**Figure 4:**
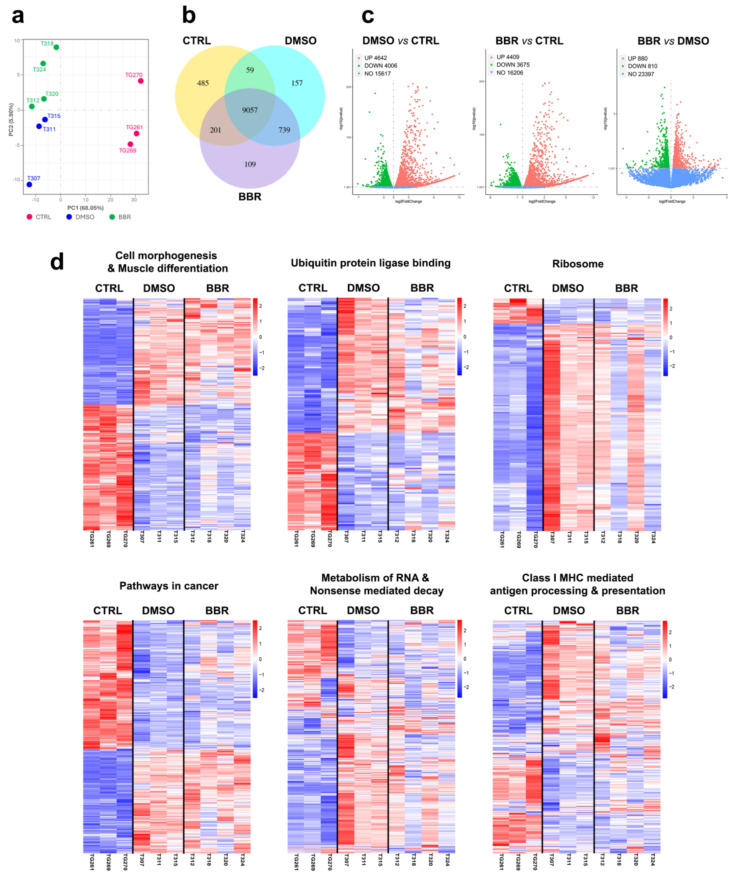
Transcriptomic analysis of TA muscle. (**a**) Principal component analysis showing considerable transcriptional separations of biological replicates between CTRL (*n* = 3), DMSO (*n* = 3), and BBR (*n* = 4) groups. (**b**) Venn diagram presents the number of genes uniquely co-expressed within each animal group, with overlapping regions showing the gene numbers co-expressed in two/three groups. (**c**) Volcano scatterplots indicate distribution of differentially expressed genes (DEGs) between 2 specific mouse groups, with each dot representing a gene (red = upregulated, green = downregulated, and blue = unchanged), x-axis showing fold-change in gene expression, and y-axis showing the statistical significance of the differences. (**d**) Heatmaps clustering analysis of gene expression in key terms/pathways selected from GO, KEGG, and Reactome enrichment (Supplementary Figure S1) are displayed. The threshold for significant enrichment was set with adjusted *p* < 0.05. The z score calculated using normalized expression values for each gene (each row) is depicted. Red color indicates genes with high expression levels and blue color indicates genes with low expression levels.

To understand why BBR downregulated DUX4 mRNA and protein expression by almost 50% of the induced level but had a marginal effect on muscle functionality and histopathology, we conducted an extensive transcriptomic analysis using RNA extracted from TA muscle. The principal component analysis on gene expression confirmed that biological replicates of the DMSO group dispersed from the BBR group and greatly dispersed from CTRL samples (Figure 4a). Among the approximately 25,000 genes identified (Supplemetary Table S1), over 9000 genes were co-expressed between the three animal groups (Figure 4b). Additional analysis on differentially expressed genes (DEGs) using DESeq2 R package identified 4642 or 4409 genes that were upregulated and 4006 or 3675 genes that were downregulated in the DMSO or BBR samples, compared to CTRL values, respectively. BBR treatment upregulated 880 while it downregulated 810 genes, compared to DMSO administration (Figure 4c). Differences in gene expression between each pair of animal groups were further clarified through genome ontology (GO), Kyoto encyclopedia of genes and genomes (KEGG), and Reactome enrichment analyses. Consistent with previous studies [22,31,32,33], we detected significant DUX4-mediated modifications (adjusted *p* < 0.05) in numerous important cellular pathways, including those involved in muscle cell development, protein homeostasis, RNA metabolism, and immune response. Details of the enrichment analyses are reported in Supplementary Figure S1 and Supplementary Tables S2–S4. We then performed heatmap clustering analysis of the most significant GO terms for cell morphogenesis and muscle differentiation (GO:0022604+GO:0042692), and ubiquitin and ubiquitin-like protein ligase binding (GO:0044389+GO:0031625), plus KEGG and Reactome pathways with the greatest enrichment of DEGs, including ribosome (KEGG mmu03010), pathways in cancer (KEGG mmu05200), metabolism of RNA and nonsense-mediated decay (Reactome R-MMU-8953854+R-MMU-927802), and class I MHC-mediated antigen processing and presentation (Reactome R-MMU-983169). As shown in Figure 4d (and more details in Supplementary Table S5), gene expression in the examined heatmaps was highly different between the CTRL and DMSO groups. BBR treatment induced a shift in the expression towards the CTRL properties, with pronounced effects seen in ribosome, metabolism of RNA and nonsense-mediated decay, and class I major histocompatibility complex (MHC)-mediated antigen processing and presentation pathways. 

## 3. Discussion

In this study, we demonstrated that systemic BBR treatment effectively inhibited inducible *DUX4* expression at both the mRNA and protein levels in one of the most utilized FSHD mouse models, ACTA1-MCM/FLExDUX4. This finding corroborates our previous research conducted on C57BL/6 mice, which involved an intramuscular delivery of exogenous *DUX4* using a viral vector system [20]. BBR-mediated suppression of *DUX4* resulted in decreased mRNA levels of murine DUX4 targets and a partial restoration of several critical cellular pathways affected by DUX4. These alterations led to an improvement in forelimb muscle strength, but they did not provide further benefits on other muscle functions, muscle mass, and histopathology of treated mice.

Transcriptomics analysis of TA muscle samples revealed positive effects of BBR on the expression of genes associated with RNA metabolism, specifically in nonsense-mediated decay, as well as in protein homeostasis, including protein translation and ubiquitination-mediated protein degradation, and in innate immune response. These cellular processes are dysregulated in FSHD [32,33,34] and are believed to be controlled by DUX4, either to evade its degradation or enhance its toxicity [35]. Hence, alleviating these processes towards healthy control values can be attributed to the direct suppression of *DUX4* by BBR. Additionally, BBR’s known properties in promoting protein degradation and enhancing innate defense mechanism, as seen in other applications [36,37], could further contribute to these beneficial outcomes. Moreover, BBR has been reported to regulate inflammation [38], lipid deposition [39], apoptosis [40], oxidative stress [41], and hypoxia [42], all of which are misregulated in FSHD [6,7,8,9,43], thus offering additional advantages as a treatment for the disease. However, the precise mechanisms underlying BBR’s multiple activities remain unclear, raising concerns about its specificity in targeting *DUX4*. As speculated in our previous publication [20], these effects might be linked to the known activity in stabilizing GQ structures of the compound, but more studies are needed for clarification of BBR’s activities, particularly in this mouse model of FSHD. 

Intriguingly, despite downregulating *DUX4* expression by approximately 50% of the induced level and enhancing grip strength performance, BBR was ineffective in improving body-wide muscle mass, other muscle functions, and muscle histopathology. This outcome contrasts with previous results we and other researchers have reported using antisense therapeutic approaches [26,31,44,45], involving specifically designed antisense oligonucleotides to target the open reading frame of the *DUX4* transcript [31], or key elements in the *DUX4* mRNA, i.e., the polyadenylation signal and the cleavage site [26]. In these studies, a similar level of *DUX4* knockdown as obtained here led to significant improvements not only in grip strength, but also in in situ muscle force, treadmill performance, and muscle histological features, although the therapeutic benefits observed were inconsistent. For example, Bouwman et al. [31] achieved similar level of *DUX4* suppression after administering antisense molecules via subcutaneous delivery and noted improvements in treadmill running distance and muscle histopathology, but they did not detect significant enhancement in grip strength and hanging rid performance or muscle mass. The disparity between our findings here and those of past studies could be attributed to differences in the dosing strategy of TMX and the age of animals. TMX is employed to activate DUX4, inducing FSHD-like pathology at various severities depending on the dosage [22]. Additionally, *DUX4* expression, even at leaky low levels in non-induced mice, accumulates overtime, leading to progressive negative impacts on muscle molecular alterations and phenotypes [46,47]. Therefore, with no overt changes in body mass and in treadmill-mediated exhaustion and with a slight increase in muscle regeneration observed in DMSO-injected mice compared to the healthy control, the model we used here apparently has a less severe phenotype than those in previous experiments. This is likely because the mice were one month younger, as likewise reported in [31]. Another possible reason can be due to the inherent variability in *DUX4* expression and pathologies in pre-clinical murine models of FSHD [22,46], as similarly seen in FSHD patients [48], which, taken together, might reduce the effects of BBR. Despite these unexpected results, our findings suggest that assessing therapeutic outcomes solely based on DUX4 inhibitory efficacy may overestimate the treatment potential, highlighting the importance of evaluating its impact on muscle functionality and histopathology for a more accurate assessment.

Consequently, further optimization of BBR’s therapeutic efficacy is warranted, which could involve adjustments to the dosing regimen, the delivery route, or the treatment length. Tan et al. [49] have reported that BBR peaked in muscle 12 h after oral delivery and remained stable for up to 48 h in rats. However, the level of BBR detected in muscle was about 7% and 36% of the levels found in the liver and kidney, respectively, where BBR was most concentrated. This implies that with more frequent dosing or increased dosages, BBR may accumulate better in muscle and exert its activity more effectively. It is expected that longer-term treatments will additionally provide more substantial benefits. Previous studies have demonstrated that mice can tolerate BBR at 5 mg/kg/day through IP injection [39] or up to 10.4 g/kg via oral administration [50]. While the latter delivery route is more relevant to clinical application, higher dosages are required due to poor gastrointestinal absorption of BBR. Indeed, clinical trials for other diseases, such as cancer, diabetes, neurological conditions, immune thrombocytopenia, and rheumatoid arthritis (https://clinicaltrials.gov/ct2/results?cond=&term=berberine&cntry=&state=&city=&dist=, accessed on 15 June 2024), have utilized oral doses of BBR in a range of 300–500 mg/dose, taken two or three times daily. Since this study represents the first use of BBR, as well as the first pharmacological intervention in this FSHD-like mouse model, further research on BBR’s absorption, metabolism, secretion, and other pharmacological properties in muscle is essential. These investigations will provide crucial information on potential applications of BBR for FSHD treatment. Given that BBR has shown no significant toxicity concerns in clinical trials for up to three years, it is envisaged that if its therapeutic effect is further validated in vivo, the pathway of BBR to clinical trials for FSHD could progress rapidly. Moreover, the therapeutic potential of BBR could be enhanced through chemical modifications to improve its bioavailability [51] and GQ-stabilizing efficacy [52], or through innovative delivery of the compound using self-microemulsifying or nanoparticle-based delivery systems [53]. 

In summary, our study confirms that BBR suppresses *DUX4* expression in the most-used FSHD-like murine model, resulting in an improvement in forelimb muscle strength and several ameliorations in DUX4-related cellular processes. Nevertheless, further optimizations of the dosing regimen, delivery route, and pharmacological properties are necessary to leverage the therapeutic potential of BBR and facilitate its translation into clinical settings for FSHD. 

## 4. Materials and Methods

### 4.1. Animals 

FLExDUX4 (JAX 028710) and ACTA1-MCM (JAX 025750) mice were purchased from The Jackson Laboratory (Bar Harbor, ME, USA). The FLExDUX4 colony was maintained as homozygous for Gt(ROSA)26Sortm1.1(DUX4*)Plj while the ACTA1-MCM colony was maintained as hemizygous for Tg(ACTA1-cre/Esr1*)2Kesr on a C57BL/6J background. The tamoxifen (TMX)-inducible double-transgenic model, ACTA1-MCM/FLExDUX4, was generated by crossbreeding ACTA1-MCM males with FLExDUX4 females. Genotyping was performed using DNA isolated from the murine ear notches based on The Jackson Laboratory’s established protocols. Mice were bred in a minimal disease facility at Royal Holloway University of London and kept under a standard 12-h light/dark cycle with free access to food and water. Animal welfare was maintained according to the UK Home Office’s Code of Practice for the Housing and Care of Animals Bred, Supplied, or Used for Scientific Purposes. Due to sex specific-DUX4 pathology of the ACTA1-MCM/FLExDUX4 model [25], only males were used in this study and littermates were randomized between experimental groups. 

### 4.2. Study Design 

The animal experiment involved 2 groups of male ACTA1-MCM/FLExDUX4 mice and 1 group of male ACTA1-MCM mice. All ACTA1-MCM/FLExDUX4 mice received the first TMX dose of 2.5 mg/kg/injection via intraperitoneal (IP) delivery at 12-weeks old (considered as week 1 of the study) and the same TMX dosage after 2 additional weeks. TMX was prepared as described previously [21] and diluted in warmed sterile corn oil to 1 mg/mL prior to use. ACTA1-MCM/FLExDUX4 mice were further IP injected with either 10 mg/kg/injection of berberine (BBR, *n* = 8), dissolved in a final concentration of 5% (*w*/*v*) DMSO, or volume-matched 5% (*v*/*v*) DMSO (*n* = 7), 2 days after the first TMX administration. ACTA1-MCM mice (*n* = 4) received volume-matched saline, considered as a healthy control (CTRL). Reagents were purchased from Sigma-Aldrich (Dorset, UK). Berberine, DMSO, or saline was readministered every 2 or 3 days thereafter for a total of 8 injections. A treadmill exhaustion test was conducted 1 week prior to the first TMX injection and repeated 4 days after the last injection. A grip strength test was performed on the same day of, but before, the last treadmill exhaustion test. In situ muscle force measurement and subsequent tissue collection were performed 1 week after the last injection. Bodyweight was recorded prior to each injection and functional test. Mice were kept under isoflurane-induced anesthesia (3% in 100% O2, *v*/*v*) during injection and were continuously monitored until they fully recovered. 

### 4.3. Functional Tests 

Treadmill exhaustion tests were performed on a Treadmill Simplex II system (Columbus Instruments, Columbus, OH, USA) with adjusted 15 °C inclination as described previously [26]. The total running time was recorded and displayed as the level of fatigue resistance, calculated as a percentage of the time recorded in the initial test. Forelimb muscle force was assessed using a commercial grip strength monitor (Linton Instrumentation, Norfolk, UK) as mentioned in [54]. Briefly, mice were held by the tail, allowed to grasp a metal mesh attached to a force transducer with their forelimbs, and then were gently pulled backwards until they released the grip. Measurements were performed 4 times per mouse, with 10 s elapsed between each time. The maximal force recorded was expressed as mN per gram of the bodyweight scored at the assessment. Tibialis anterior (TA) muscle strength was assessed with in situ muscle force measurement as detailed in [26] while mice were under terminal anesthesia, induced by a mixture of 10 mg/mL dolethal (Vetoquinol, Towcester, UK) and 15 µg/mL buprenodale (Dechra, Shrewsbury, UK) at 6 mL/kg. Data were recorded and analyzed using Dynamic Muscle Control and Analysis Software (Aurora Scientific, Aurora, ON, Canada). All isometric measurements were obtained at an initial length at which a maximal tension was recorded during the tetanus. Responses to tetanic stimulations at increased pulse frequencies from 10 Hz to 180 Hz were recorded and the maximal force was determined. 

### 4.4. Post-Mortem Tissue Processing

From each mouse, the diaphragm (DIA), quadriceps (QUAD), TA, and triceps muscles were dissected and weighed after fat/connective tissue was trimmed off. Muscles from one side of the body were frozen immediately in liquid nitrogen for subsequent molecular analyses. Contralateral muscles were embedded in optimal cutting temperature (OCT) medium (VWR, Lutterworth, UK) and frozen in liquid nitrogen-cooled isopentane (Sigma-Aldrich, Dorset, UK) for histological analyses. DIA muscle was also longitudinally divided into 2 halves. One half was frozen immediately in liquid nitrogen while the other half was prepared as described in [55] for histological analyses. OCT-embedded frozen muscles were cryosectioned on an OTF5000 cryostat (Bright, London, UK) at 10 µm thickness for 10 serial levels through the muscle length, and transverse sections were collected onto SuperFrost slides (VWR, Lutterworth, UK). 

### 4.5. RNA Extraction, cDNA Synthesis, and RT-qPCR Quantification for DUX4 and Relevant Genes 

Total RNA from liquid nitrogen snap-frozen TA muscles was isolated using an RNeasy Fibrous Tissue kit (QIAGEN, Manchester, UK) following the manufacturer’s instructions. Samples were homogenized in the lysis buffer provided with the kit on a TissueLyser II (QIAGEN, Manchester, UK) at 25 Hz for 2 min. One microgram of RNA was reverse transcribed using a QuantiTect reverse transcription kit (QIAGEN, Manchester, UK). Ten nanograms of diluted DNA in qPCR water (Roche, Welwyn Garden City, UK) were then amplified using LightCycler480 SYBR Green Master I kit (Roche, Welwyn Garden City, UK) according to the manufacturer’s instructions, with each sample prepared in triplicates. qPCR reactions were run on a LightCycler480 System, initialized at 95 °C for 5 min, followed by 45 cycles at 95 °C for 15 s, 60 °C for 15 s, and 72 °C for 15 s. Relative quantification for *DUX4* and DUX4-related genes was performed against corresponding housekeeping gene, *Gapdh*. Data are shown as fold-change compared to the DMSO values obtained in the same way. Primers, detailed in [26], were purchased from Integrated DNA Technologies (Leuven, Belgium).

### 4.6. Protein Extraction, Protein Assay, and DUX4 Immunoblotting 

Snap-frozen TA muscles (as described above) were homogenized in lysis buffer (0.15 M NaCl, 0.05 M HEPES, 1% (*v*/*v*) NP-40, 0.5% (*w*/*v*) sodium deoxycholate, 0.1%(*w*/*v*) SDS, 0.01 M EDTA; reagents were purchased from Sigma-Aldrich, Dorset, UK) containing protease inhibitors (Roche, Welwyn Garden City, UK) at 25 Hz for 2 min on a TissueLyser II (QIAGEN, Manchester, UK). Following centrifugation at 14,000× *g*, 10 min, and 4 °C, the supernatant was transferred to fresh pre-chilled 1.5 mL tubes. The total protein content was quantified using a DC Protein Assay (Bio-Rad, Watford, UK) following the manufacturerc’s instructions. Fifty micrograms of protein samples were denatured at 70 °C for 10 min and then resolved on a 4–12% Bis-Tris NuPage gel (Life Technologies, Paisley, UK). An NEB color pre-stained protein ladder (Hitchin, UK) was used as a size standard. The gel was run at 150 V for 1.5 h, and was subsequently transferred to HyBond nitrocellulose membrane (GE Healthcare, Hatfield, UK) at 30 V for another 1.5 h. The membrane was incubated in blocking buffer, containing 5% (*w*/*v*) skimmed milk, 1× PBS, and 0.2% (*v*/*v*) Tween-20, for 1 h prior to overnight incubation at 4 °C with mouse anti-vinculin antibody (SAB4200080-200, 1:10,000, Sigma-Aldrich, Dorset, UK) and rabbit anti-DUX4 antibody (ab124699, 1:1000, Abcam, Cambridge, UK). Subsequent incubation with goat anti-mouse IRDye680LT and goat anti-rabbit IRDye800CW (1:10,000, LI-COR Biosciences, Cambridge, UK) was carried out for 1 h, at room temperature. The blot was visualized on an Odyssey Infrared Imaging System (LI-COR Biosciences, Cambridge, UK). Densitometric analysis of vinculin- and DUX4-positive bands was performed using Image Studio software, v5.2 (LI-COR Biosciences, Cambridge, UK). The values of DUX4 intensity were normalized to the values of corresponding vinculin intensity and expressed as fold-change in the values of the DMSO group obtained in the same way. 

### 4.7. Immunohistochemistry 

Frozen TA muscle sections were fixed in ice-cold acetone for 10 min and blocked in 1% (*w*/*v*) BSA, 1% (*v*/*v*) goat serum, 0.1% (*v*/*v*) Triton X-100, and 1× PBS for 1 h at room temperature. Subsequent incubation with rat anti-laminin (L0663, 1:1000, Sigma-Aldrich, Dorset, UK) and rabbit anti-collagen VI (ab6588, 1:300, Abcam, Cambridge, UK) antibodies was carried out for 2 h at room temperature, followed by 1 h incubation with goat anti-rat AlexaFluor568 and goat anti-rabbit AlexaFluor488 antibody (1:500, Life Technologies, Paisley, UK). Nuclei were stained with 1 µg/mL DAPI in 1× PBS for 15 min. Slides were mounted in a Mowiol 4-88. Reagents were purchased from Sigma-Aldrich (Dorset, UK) unless stated otherwise. Six random images from the largest mid-belly of each muscle section were captured at a magnification of ×100. Images were acquired on an Axio Observer D1 fluorescence microscope using an AxioCam MR3 (Zeiss, Cambridge, UK).

### 4.8. Histological Analyses

The cross-sectional area (CSA) of TA muscles was mathematically approximated by dividing the muscle mass by the optimum fiber length and the density of mammalian muscle as described in TREAT-NMD SOP DMD_M.2.2.005. The myofiber perimeter was identified via laminin staining. Subsequent quantification of the total fiber number, the number of centrally nucleated fibers (CNFs), and the minimal Feretc’s diameter of individual myofibers were automatically scored using Fiji/MuscleJ software, v1.53c (National Institutes of Health, Bethesda, MD, USA), as described in [56]. Automatic analyses of the frequency distribution of the minimal Feret’s diameter were carried out using GraphPad Prism8 software (Boston, MA, USA). The area positive with collagen VI was semi-automatically measured with Fiji/MuscleJ. Fibrotic area was calculated as the percentage of the total area of the muscle CSA. 

### 4.9. RNA-seq Analyses

Total RNA content extracted from TA muscles, as described above, was sent to Novogene (Cambridge, UK) for transcriptomics analyses. Messenger RNA was first purified using poly-T oligo-attached magnetic beads. After fragmentation, the first strand cDNA was synthesized using random hexamer primers, followed by the second strand cDNA synthesis using either dUTP for directional library or dTTP for non-directional library. The library was checked with Qubit and qPCR for quantification and bioanalyzer for size distribution detection. Quantified libraries will be pooled and sequenced on Illumina platforms. The clustering of the index-coded samples was performed according to the manufacturer’s instructions. After cluster generation, the library preparations were sequenced on an Illumina NovaSeq PE150 platform, and paired-end reads were generated. All the downstream analyses were based on the clean, high-quality data obtained by removing reads containing adapters, reads containing ploy-N, and low-quality reads from raw data. Paired-end clean reads were then aligned to a reference genome using Hisat2, v2.0.5. The reads numbers mapped to each gene were counted using featureCounts v1.5.0-p3. The FPKM, Fragments Per Kilobase of transcript sequence per Millions base pairs sequenced, of each gene was then calculated based on the length of the gene and the reads count mapped to this gene. Differential expression analysis of two groups was performed using the DESeq2 R package (1.20.0). The resulting *p* values were adjusted using the Benjamini and Hochberg’s approach for controlling the false discovery rate. Genes with an adjusted *p* < 0.05 found via DESeq2 were assigned as differentially expressed. Enrichment analyses of differentially expressed genes (DEGs) were implemented using the clusterProfiler R package, in which gene length bias was corrected. Gene ontology (GO, http://www.geneontology.org/, accessed on 15 June 2024) terms, Kyoto Encyclopedia of Genes and Genomes (KEGG, http://www.genome.jp/kegg/, accessed on 15 June 2024), and Reactome (https://reactome.org/, accessed on 15 June 2024) pathways with corrected *p* < 0.05 were considered significantly enriched by DEGs. Data visualization was obtained using Novogene’s innovative software, NovoSmart, which is developed based on R Shiny. 

### 4.10. Statistical Analysis

Data were analyzed using GraphPad Prism8 software (Boston, MA, USA) and are shown as the means ± S.E.M. Error bars represent the S.E.M; “n” refers to the number of mice per group. All data passed the normality Shapiro–Wilk test, which is the most powerful test among four common normality tests especially for small sample size (3 ≤ n ≤ 5000) [57]. Comparisons of statistical significance were further assessed with the Student’s *t* test and one-way or two-way ANOVA followed by Tukey’s post hoc test, as detailed in figure legends. All functional tests and histological analyses were performed in a blind manner. 

## Data Availability

The data presented in this study are available upon request from the corresponding author. The data are not publicly available due to further development of the work.

## Supplementary Materials

The following supporting information can be downloaded at: https://www.mdpi.com/article/10.3390/ijms25136994/s1.
