# Supplementary material for: Systemic Pharmacotherapeutic Treatment of the ACTA1-MCM/FLExDUX4 Preclinical Mouse Model of FSHD
Source: Int J Mol Sci. 2024 Jun 26;25(13):6994. doi: 10.3390/ijms25136994 (PMC11241187; doi:10.3390/ijms25136994)
Supplement: Supplementary file 1 [file ijms-25-06994-s001.zip › Supplementary Figure S1.pdf]

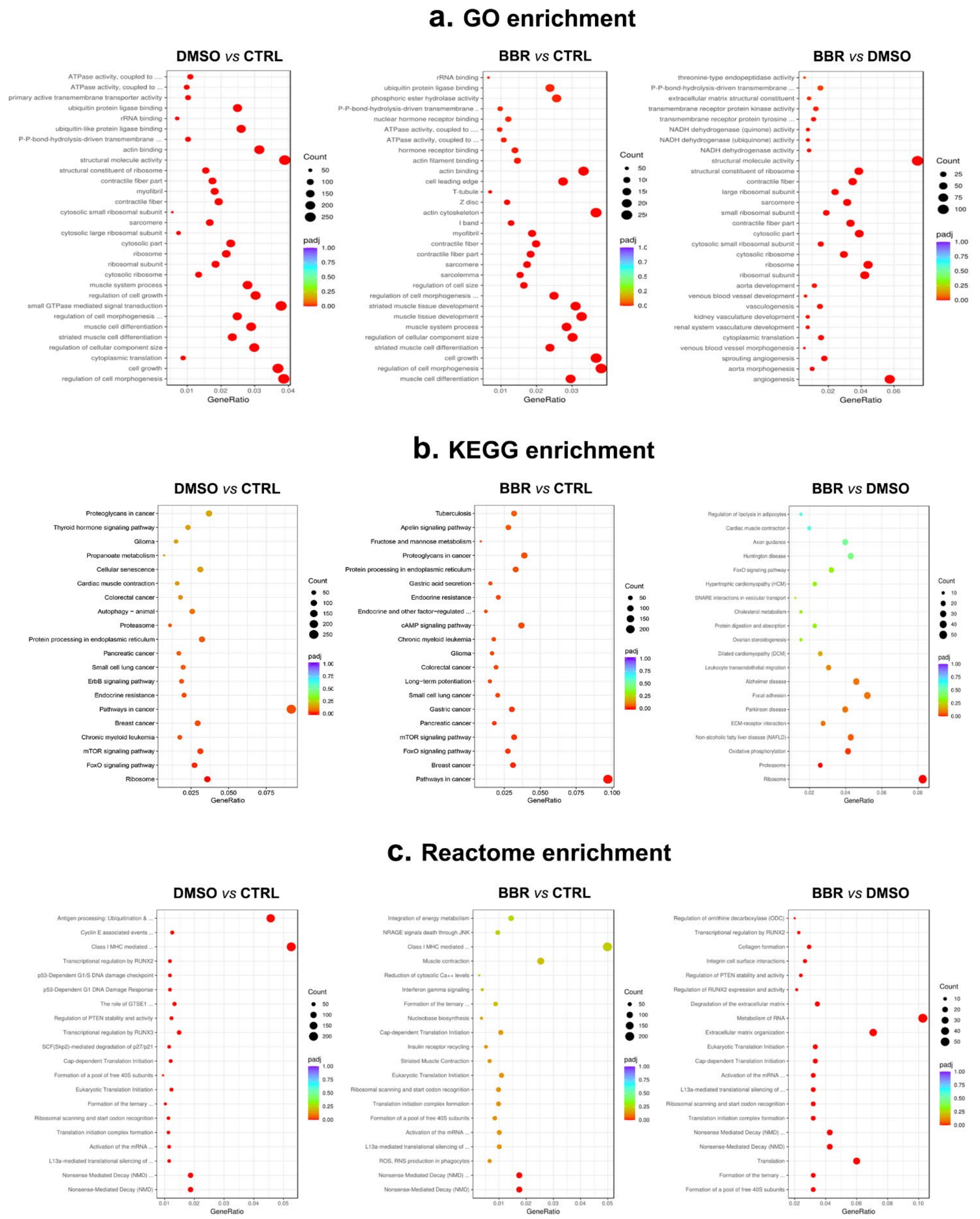


**Supplementary Figure 1. Gene enrichment analysis of TA muscle.** RNA from TA muscle of CTRL (*n*=3), DMSO (*n*=3) and BBR (*n*=4) groups underwent transcriptomic analysis. **(a)** Thirty most significant terms from GO enrichment analysis of DEGs between 2 specific groups are shown in order of 10 molecular function, 10 cell component and 10 biological process terms (top to bottom). The most significant (or most relevant if not significant) 20 pathways from **(b)** KEGG or **(c)** Reactome enrichment analysis of DEGs between 2 animal groups are displayed. The point size represents the number of genes annotated to a particular term or pathway while the color from red to purple represents the significant level of the enrichment, and the abscissa represents the ratio of the DEG number on the term or pathway to the total number of DEGs. The threshold for significant enrichment was set with adjusted $p<0.05$.
